# Medical Studies and Mnemonics

**Published:** 1916-08-05

**Authors:** 


### Medical Studies and Mnemonics.

Many of us can recall to mind the old rhyme used by sailors in studying navigation, part of which runs

Green to green and red to red,
Perfect safety, go ahead, and the many metrical aids to memory of school days. We do not, however, remember anything so exhaustive in character (in more senses than one) as the "Mnemonic of Inorganic Analysis," which appears in the July number of that bright and witty little journal the *Magazine of the London (Royal Free Hospital) School of Medicine for Women.* We are told that

If you will learn this little rhyme
You'll pass First Medical—in time!
Given a salt you do not know,
Start analysing, as below.

Then follow about 130 lines of verse, those portions describing confirmatory tests being placed in brackets. Of the tests we select a specimen to illustrate the humour and ingenuity of the whole:

Add $H_2S$—him with the smell
Precipitates Group II., well, well!
Sn, Sb, also As
Dissolve in Amo. Sulph. I guess.
Add nitric to the residue,
Hg alone remains with you.

And the following confirmatory test:

To orig. sol. add pot. ferro.,
The ferrous green, now white will go.
If sol. was red, it was ferric;
Pot. ferro. shows the men we lick.

A very necessary footnote explains that Prussian blue is indicated by the last few words. We congratulate "$(E.D.F.)^2$" on her clever effort, but whether it would be more useful as a recitation at a students' smoking concert (but are there such things in a school for women?) or as a mnemonic we do not dare to decide.

